# sRNAanno—a database repository of uniformly annotated small RNAs in plants

**DOI:** 10.1038/s41438-021-00480-8

**Published:** 2021-03-01

**Authors:** Chengjie Chen, Jiawei Li, Junting Feng, Bo Liu, Lei Feng, Xiaoling Yu, Guanliang Li, Jixian Zhai, Blake C. Meyers, Rui Xia

**Affiliations:** 1 State Key Laboratory for Conservation and Utilization of Subtropical Agro-Bioresources, South China Agricultural University, Guangzhou, China; 2 Guangdong Laboratory for Lingnan Modern Agriculture, South China Agricultural University, Guangzhou, China; 3 Key Laboratory of Biology and Germplasm Enhancement of Horticultural Crops in South China, Ministry of Agriculture and Rural Affairs, South China Agricultural University, Guangzhou, Guangdong 510640 China; 4 Guangdong Litchi Engineering Research Center, College of Horticulture, South China Agricultural University, Guangzhou, Guangdong 510640 China; 5 Department of Biology & Institute of Plant and Food Science, Southern University of Science and Technology, Shenzhen, Guangdong 518055 China; 6 Donald Danforth Plant Science Center, Saint Louis, MO 63132 USA; 7 Division of Plant Sciences, University of Missouri–Columbia, Columbia, MO 65211 USA

**Keywords:** Non-coding RNAs, Gene silencing

## Abstract

Small RNAs (sRNAs) are essential regulatory molecules, and there are three major sRNA classes in plants: microRNAs (miRNAs), phased small interfering RNAs (phased siRNAs or phasiRNAs), and heterochromatic siRNAs (hc-siRNAs). Excluding miRNAs, the other two classes are not well annotated or available in public databases for most sequenced plant genomes. We performed a comprehensive sRNA annotation of 143 plant species that have fully sequenced genomes and next-generation sequencing sRNA data publicly available. The results are available via an online repository called sRNAanno (www.plantsRNAs.org). Compared with other public plant sRNA databases, we obtained was much more miRNA annotations, which are more complete and reliable because of the consistent and highly stringent criteria used in our miRNA annotations. sRNAanno also provides free access to genomic information for >22,721 *PHAS* loci and >22 million hc-siRNA loci annotated from these 143 plant species. Both miRNA and *PHAS* loci can be easily browsed to view their main features, and a collection of archetypal *trans-acting siRNA 3* (*TAS3*) genes were annotated separately for quick access. To facilitate the ease of sRNA annotation, sRNAanno provides free service for sRNA annotations to the community. In summary, the sRNAanno database is a great resource to facilitate genomic and genetic research on plant small RNAs.

## Introduction

Small RNAs (sRNAs) are essential regulatory molecules in plants. With the rapid development of deep-sequencing technologies and bioinformatics, sRNAs have been characterized in an increasing number of plant species, leading to the generation of large amounts of next-generation sequencing (NGS) data. A large number of raw NGS sRNA data have been deposited in public databases, such as the Sequence Read Archive (SRA), Gene Expression Omnibus (GEO) and European Nucleotide Archive (ENA) databases. MicroRNAs (miRNAs) are the most well-studied class of sRNAs in plants. To date, miRBase is the primary repository and online database for annotated miRNAs^1^. As a routine practice in the research community, the annotated miRNAs of a species are required to be deposited into miRBase before publication; i.e., author submission is the primary data source of the database. This process makes it hard to maintain a high quality of annotated miRNAs deposited in miRBase because of the variable stringency of the criteria, controlled by the submitting authors who are responsible for miRNA annotations. In other words, rather than the developers or maintainers of miRBase, quality control is more reliant on the authors, reviewers and editors, who likely have a different understanding of the criteria of miRNA annotation. Therefore, the variable reliability of annotated miRNAs in miRBase is of great concern to the community^2^.

In addition to miRNAs, other types of sRNAs exist in plants, including phased small interfering RNAs (phased siRNAs or phasiRNAs) and heterochromatic siRNAs (hc-siRNAs). phasiRNAs have recently emerged as critical regulatory molecules in nearly all aspects of plant growth and development^3^. They are widely present in plants—from algae to angiosperms. However, compared to miRNAs, phasiRNAs are much less studied and are not well annotated for most of the plants species whose genome has been fully sequenced^4,5^. To date, there is no public database of annotation information of plant phasiRNAs, hindering the application of already annotated phasiRNA information. Heterochromatic siRNAs are the most abundant class of sRNAs in plants, and they usually play roles related to DNA methylation, which is a process important for transcriptional regulation. Although we know their functional importance, thorough annotations of hc-siRNA-generating genomic regions (hc-siRNA loci) are lacking for most plant genomes.

In this study, we conducted extensive sRNA annotations of 143 plant species whose genome has been fully sequenced and for which at least one sRNA deep-sequencing data set is available in public databases. The annotations include all three sRNA classes: miRNAs, phasiRNAs, and hc-siRNAs. To achieve high confidence for miRNA annotations, we applied a set of uniform criteria adopted from the recently updated rules^2^. For phasiRNA annotations, a *p* value-based approach established by our group was used for annotations of loci, yielding 21-nt phasiRNAs (21-*PHAS*) or 24-nt phasiRNAs (24-*PHAS*)^6,7^. We also developed an algorithm based on sequence repetitiveness for the accurate annotations of loci generating hc-siRNAs, given their primary feature of generation from repetitive genomic regions. In total, we annotated 24,630 miRNA hairpins or precursors, 22,721 *PHAS* loci (18,239 21-*PHAS* and 4,482 24-*PHAS*), and 22,404,950 hc-siRNA loci. All these results have been deposited in an online database of sRNA annotations (sRNAanno) for open access. This database is a great resource for research on plant sRNAs.

## Database content

### sRNAanno database

**Fig. 1:**
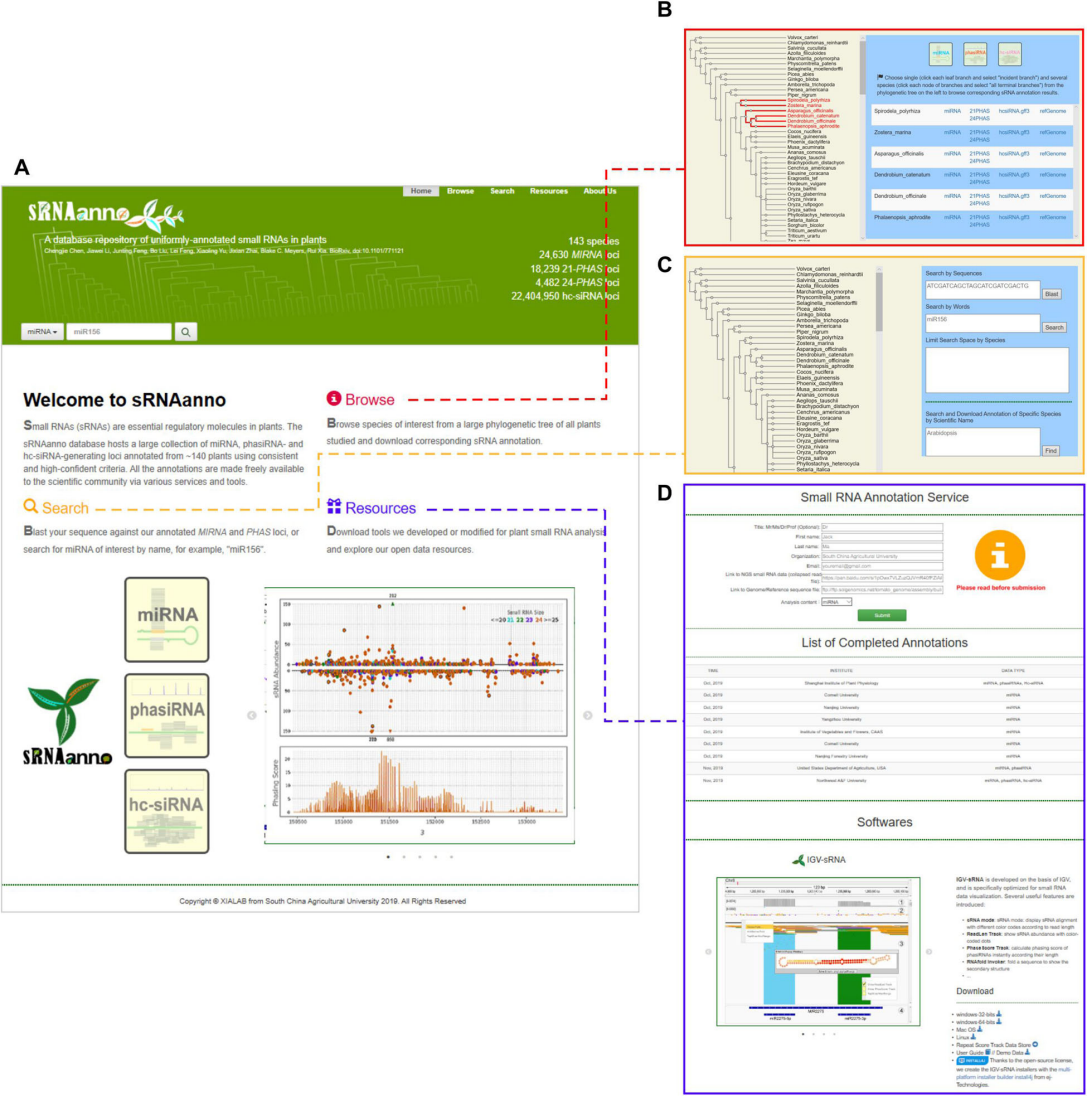
Overview of the sRNAanno database. **A** Screenshots of the HOME page and subpages of the three main functions: BROWSE (**B**), SEARCH (**C**), and RESOURCES (**D**)

Small RNA annotation of three major sRNA classes was performed for 143 plant species, and an online database (sRNAanno, www.plantsRNAs.org) was constructed to store all the annotation results for easy and quick public access. There are three major functions within sRNAanno:

**BROWSE**, for browsing annotation results, **SEARCH**, for searching for certain information, and **RESOURCES**, for data sharing (Fig. 1A). On the **BROWSE** page, users can select a single or several species from a large phylogenetic tree and browse or download corresponding small RNA annotation results (Fig. 1B). The **SEARCH** function includes miRNA searches by either miRNA name or sequence comparison using the BLAST function (Fig. 1C). The **RESOURCES** page provides quick access to the Small RNA Annotation Service page and other relevant data (Fig. 1D); for instance, the free software IGV-sRNA, which is designed for the exploration of sRNA data, can be download here.

### miRNA annotations

**Fig. 2:**
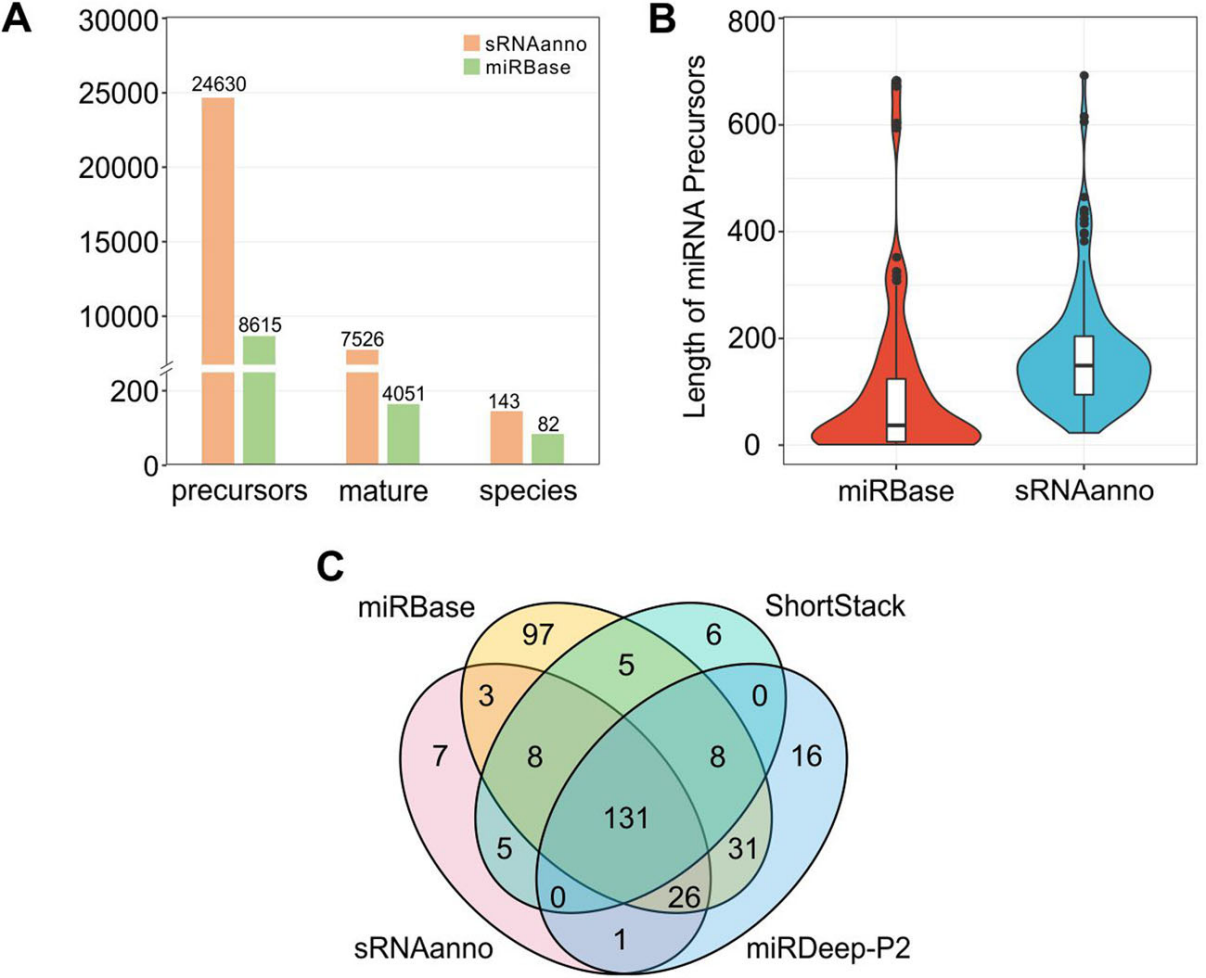
Summary of annotated miRNAs in sRNAanno. **A** Comparison of the numbers of plant species and *MIRNA* loci annotated between miRBase and sRNAanno. **B** Length of miRNA precursors in miRBase and sRNAanno. **C** Comparison of the miRNA annotation results of sRNAanno for Arabidopsis with those from three other sources/pipelines: miRBase, ShortStack, and miRDeep-P2

In this study, we aimed to conduct genome-wide annotations of plant miRNAs using a set of uniform and well-established criteria (Fig. S1), as well documented before^2,8^. To this end, we downloaded the genome sequences for nearly all species for which both their genome and their sRNA NGS data are available from public databases (such as SRA, ENA, and GEO). We found 143 plant species with corresponding sRNA data available. In total, there are 1,606 small RNA sequencing data sets, most of which are generated from well-studied model plant species, such as Arabidopsis and rice. We performed miRNA annotations of all these species and obtained 24,630 annotated hairpin precursors encoding 7,526 unique mature miRNA sequences (Fig. 2A). Compared with other public sRNA databases, like miRbase^1^, PmiREN^9^, and “Plant Small RNA Genes”^10^, sRNAanno employed more small RNA datasets, or covered many more plants, with the annotation of more *MIRNA* loci. For example, compared with the annotations in the latest release of miRBase (v22), which contains 8,615 annotated hairpin precursors from 82 plant species with 4,051 mature miRNA sequences (Fig. 2A), our annotations yielded more results in terms of not only the number of species annotated (a 1.74-fold increase) but also the number of miRNA precursors (a 2.86-fold increase) (Fig. 2A). In plants, ~24 miRNA families predominate in angiosperms^11^. To assess the completeness of miRNA annotations of a species, we compared the number of conserved miRNA families in species that have data in both sRNAanno and miRBase. We found that nearly all 45 species analyzed had a more complete list of conserved miRNAs in sRNAanno, while conserved miRNAs in 14 species were obviously incomplete in miRBase (v22) (Fig. S2). Moreover, in terms of the length distribution of *MIRNA* precursors, the length of most precursors in miRBase is much shorter (<100 bp) than that in sRNAanno (100–200 bp, Fig. 2B), which seems reasonable, as the majority of Arabidopsis *MIRNA* precursors are 100–200 bp in length^12^. Therefore, compared with miRBase (v22), sRNAanno has more complete and reliable plant miRNA data. When comparing with miRNAs deposited in miRBase or identified by other tools (miRDeep-P2 and ShortStack) within a species (using Arabidopsis and rice as examples), we found that the majority of miRNAs annotated in sRNAanno were also identified by at least one of the other tools^13,14^ (Fig. 2C and Fig. S3); only a few of them were unique to sRNAanno. Overall, we contend that our miRNA annotations are of high stringency and high confidence.

### *PHAS* locus annotation

**Fig. 3:**
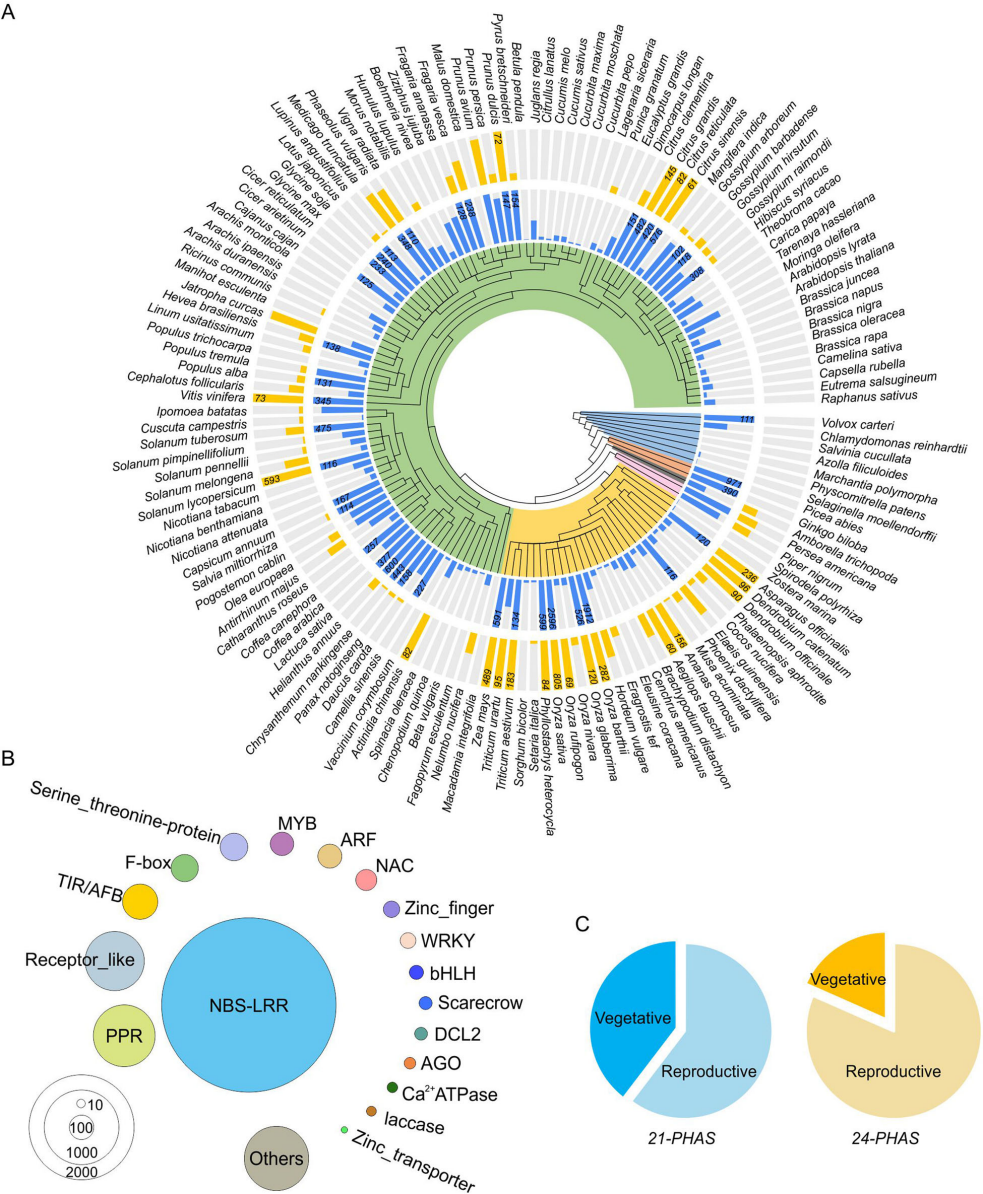
Summary of annotated *PHAS* loci in sRNAanno. **A** Twenty-one (inner circle, blue, with the maximum value set to 100) and 24 (outer circle, yellow, with the maximum value set to 50) *PHAS* loci annotated in each species. All the species are ordered according to the phylogenetic tree of APG IV (Angiosperm Phylogeny Group IV). **B** Functional classification of protein-coding 21-*PHAS* loci. The circle sizes are scaled by the number of 21 *PHAS* loci in a given gene group. **C** Tissue-specific enrichment of 21- and 24-*PHAS* loci

Phased siRNAs (phasiRNAs) are another major class of sRNAs found in plants, and these are universally present in all plants, mainly as members of the trans-acting siRNA (tasiRNA) subgroup^15^. This group is characterized by the phasing pattern of sRNAs, which exhibit an approximately head-to-tail arrangement. To date, unlike for miRNAs, there is no database of identified or reported *PHAS* genes or genomic loci, although phasiRNAs have been profiled in a large number of plants^3^. Therefore, we performed an exhaustive *PHAS* profiling of the 143 plant species for which at least one sequenced sRNA library exists. We used a well-developed *p* value-based protocol to perform *PHAS* analysis^7,16^ (Fig. S4). The cutoff of the *p* value was set to 1e-3. For analysis of 24-*PHAS* loci (generating 24-nt phasiRNA), we added an additional filter to remove repetitive sequences that usually give yield abundant 24-nt hc-siRNAs. In total, we identified 18,239 21-*PHAS* loci generating 21-nt phasiRNAs and 4,482 24-*PHAS* loci generating 24-nt phasiRNAs. In general, the number of 21-*PHAS* loci was substantially greater than that of 24-*PHAS* loci within a species (Fig. 3A). Both types of *PHAS* loci are not evenly present across species (Fig. 3A), perhaps because of the intrinsic genomic differences among plant species; for instance, plants from certain families, such as the Brassicaceae (including the model plant Arabidopsis) and Cucurbitaceae, consistently yield fewer *PHAS* loci than do other species (Fig. 3A). As reported before, 24-*PHAS* loci are noticeable more widespread in monocots but are dispersed in eudicots^7^(Fig. 3A). Other factors accounting for this uneven distribution of *PHAS* loci are likely the sampling for sRNA sequencing (tissue, stage, etc.) and sequencing technology (sequencing platforms, sequencing depth, etc.).

Protein-coding genes are a rich source of phasiRNAs. After functionally annotating the 21-*PHAS* loci and assessing their protein-coding capacity, we found that a large number of gene families produce a large number of phasiRNAs, especially for members of the gene families *NBS-LRR*, *PPR*, *Receptor-like kinase*, etc. (Fig. 3B). In particular, many transcription factor-encoding genes produce phasiRNAs, including *TIR/AFB*, *F-box*, *NAC*, *MYB*, *ARF*, *WRKY*, *zinc finger*, and *bHLH* genes (Fig. 3B). In terms of noncoding *PHAS* loci, for both 21-*PHAS* and 24-*PHAS*, most were enriched in reproductive tissues (Fig. 3C).

### Browsing miRNA and *PHAS* loci in sRNAanno

**Fig. 4:**
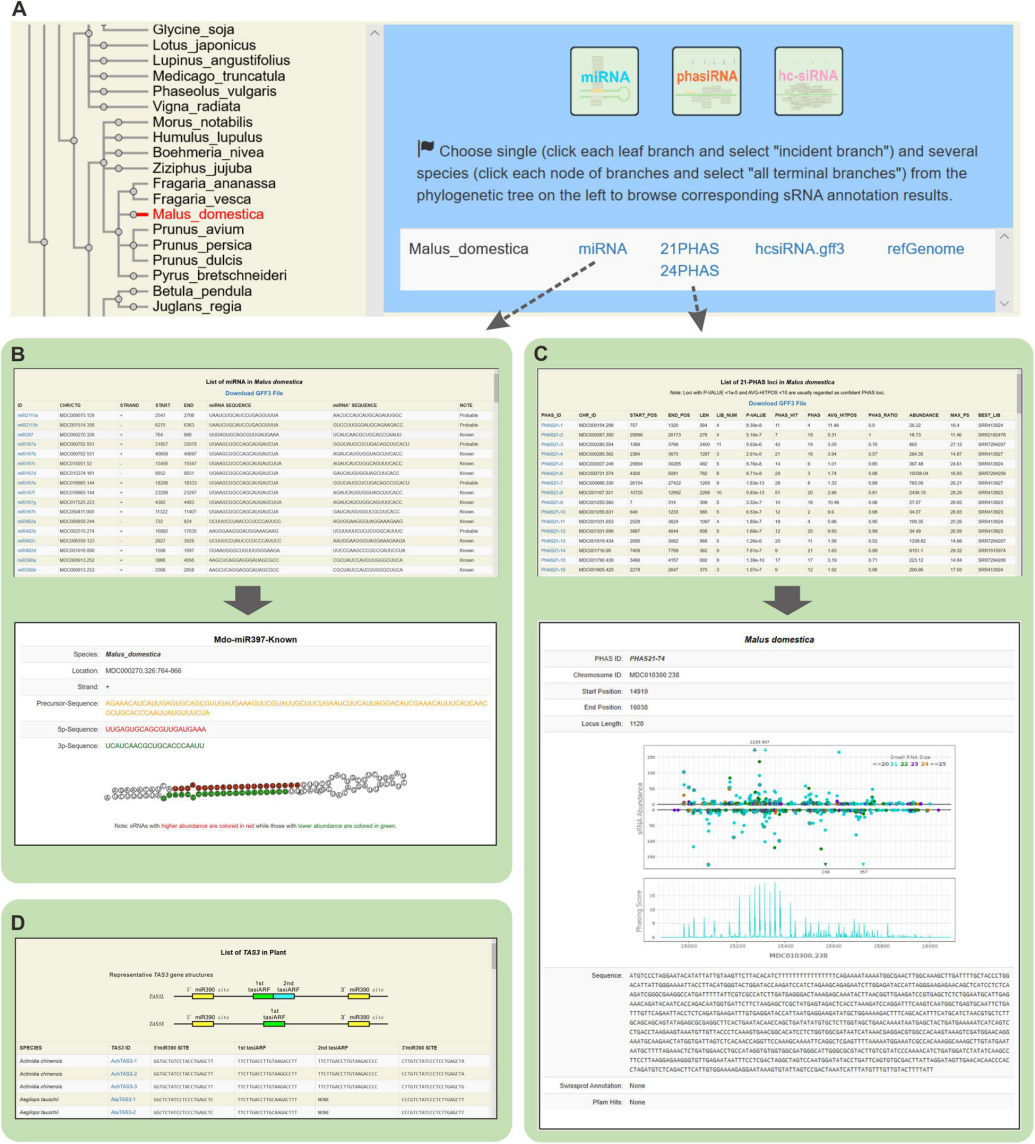
Exploration of miRNA and *PHAS* loci in sRNAanno. Demonstrative screenshots of the BROWSE page and linked pages for three main functions: Phylogenetic tree of all species in sRNAanno (**A**), miRNA list (**B**), *PHAS* loci list (**C**), and *TAS* gene list (**D**)

All the annotated miRNA and *PHAS* loci can be easily explored on the **BROWSE** page in sRNAanno (Fig. 4A). For a given species, the miRNAs are listed in a table, with the main information included, such as chromosomal coordinates, sequences of miRNAs, and miRNAs* (Fig. 4B). For each miRNA, a page of detailed information is also linked, in which a folded secondary structure can be found. For *PHAS* loci, a summary table is also provided for each species (Fig. 4C). Major features of each *PHAS* locus, such as chromosomal coordinates, *p* value, sRNA abundance, maximum phasing score, and data sources, are listed in detail. The sRNA distribution and phasing score of each *PHAS* locus are displayed on a linked page, with additional information listed, including the sequence of the *PHAS* locus, SwissProt annotations, and potential Pfam domain structure (Fig. 4C).

*TAS3* is an ancient and archetypal *PHAS* gene that is widely conserved in all terrestrial plants^15^. *TAS3* has features distinct from most *PHAS* genes: it is usually targeted at two sites by miR390 and generates one or two tasiARFs (tasiRNAs targeting *ARF* genes)^15,17^. The miR390-*TAS3*-*ARF* pathway plays essential role in the auxin signaling network. Here, we annotated 232 *TAS3* genes from our *PHAS* analysis results, and all these *TAS3* genes are collectively represented in an independent table (Fig. 4D), in which the sequences of miR390 target sites and tasiARF(s) are included. Links to the corresponding page of *PHAS* features are also provided for convenient browsing.

### hc-siRNA locus annotations

hc-siRNAs account for a major part of the plant cellular sRNA population. At present, the genomic loci generating hc-siRNAs have not been thoroughly annotated owing to their large number, variability in sRNA abundances, and complexity of their biogenesis. hc-siRNAs are generated from repeat-related sequence regions, typically transposons, and heterochromatic regions, to direct *cis* DNA methylation in plants^18^. As these regions generating hc-siRNA are also important components of genomic information for a species, we annotated hc-siRNA loci for genomes with sRNA data available according to the criteria listed in Fig. S5. Indeed, these hc-siRNA-generating loci are abundant in almost every genome (Fig. S6).

### sRNA annotation service

Small RNA data analysis using various bioinformatic software or pipelines relying on programming and command-line environments is challenging and time-consuming for most wet-lab biologists. To facilitate the ease of sRNA annotation, we are providing free service for sRNA annotations in sRNAanno. Users can upload sRNA NGS data and corresponding genome/reference sequence file to an accessible online repository (such as an FTP site) and submit download links to these files to sRNAanno on the RESOURCES page. Upon receiving the information, we will download the data files, perform the sRNA annotations, and return the annotation results to the users by email. For this service, we will maintain high confidence of users’ data and results and will not, under any circumstance, use them or release them to the public without users’ permission.

## Discussion

The new database repository of plant small RNAs described here, sRNAanno, is a repository of major types of sRNAs for >140 plant genomes. These extensive annotations were achieved by analyzing ~1,600 sRNA datasets using well-established computation pipelines with reliable and highly stringent criteria. The sRNAanno database includes miRNA annotations of ~64% more plant species than the number within miRBase, the main and most popular miRNA hub, and the number of miRNA annotations in sRNAanno is also much greater than the number in recently published databases, including PmiREN, and “Plant Small RNA Genes”^9,10^. Moreover, all the miRNAs in sRNAanno were annotated via an identical process with consistent criteria, in contrast to the variable annotation criteria used for the miRNAs in miRBase, whose annotations was conducted by different research groups with various tools^19^. Generally, we believe that the miRNA annotations in sRNAanno are more reliable than those in miRBase. However, there is no gold standard for annotations of plant miRNAs. Although there are misannotations in miRBase, there may be a certain number of bona fide miRNAs that are possibly missing in sRNAanno. Moreover, miRBase also houses miRNAs from plant species whose genome has not been sequenced or for which no publicly available sRNA data are available (for which we are unable to perform miRNA annotations). Therefore, sRNAanno is a good complement, instead of a substitute, to miRBase.

In addition to miRNAs, sRNAanno also stores information concerning genomic loci generating phasiRNAs or hc-siRNAs. Annotations of these sRNA-generating loci were conducted using high confidence settings according to the widely accepted criteria. In plants, phasiRNAs have emerged as one of the major types of sRNAs, and their targets function in a broad range of biotic and abiotic processes. For instance, phasiRNAs are abundantly produced during the reproductive stage, especially in monocots and their subgroups of grasses^15,20^. In rice, there are >2,000 *PHAS* loci generating 21-nt phasiRNAs and ~400 loci generating 24-nt phasiRNAs^21^. Although phasiRNAs, including well-known tasiRNAs, have been characterized in an increasing number of plant species, there is no public online repository of reported or annotated phasiRNAs to provide convenient and quick access to this information. Similarly, hc-siRNAs are widely present in plant cells and are well known for their connection to DNA methylation or other epigenetic modifications, but the majority of plant genomes lack good annotations of hc-siRNA loci. In this study, we performed broad annotations of phasiRNAs and hc-siRNAs in plants, and the resulting annotations stored in sRNAanno constitute a valuable resource to facilitate genomic and genetic research in plants.

## Conclusions

Thorough annotations of miRNAs, phasiRNAs, and hc-siRNAs were conducted for the genome of 143 plant species. All the annotation results are of high quality and confidence and have been deposited in the public database repository sRNAanno (www.plantsRNAs.org) for quick and convenient access. Both miRNA and *PHAS* loci can be easily browsed to view their main features. All these data and results are valuable resources facilitating research on sRNAs or related areas of plants.

## Supplementary information

Supplemental table

Supplemental Figures

Supplemental Figures

## Data Availability

All the open-access data (including all the annotated sRNA results) are available through the sRNAanno database (www.plantsRNAs.org)
