# Supplementary material for: sRNAanno—a database repository of uniformly annotated small RNAs in plants
Source: Hortic Res. 2021 Mar 1;8:45. doi: 10.1038/s41438-021-00480-8 (PMC7917102; doi:10.1038/s41438-021-00480-8)
Supplement: Supplementary file 2 — Supplemental Figures [file 41438_2021_480_MOESM2_ESM.pptx]

## Slide 1
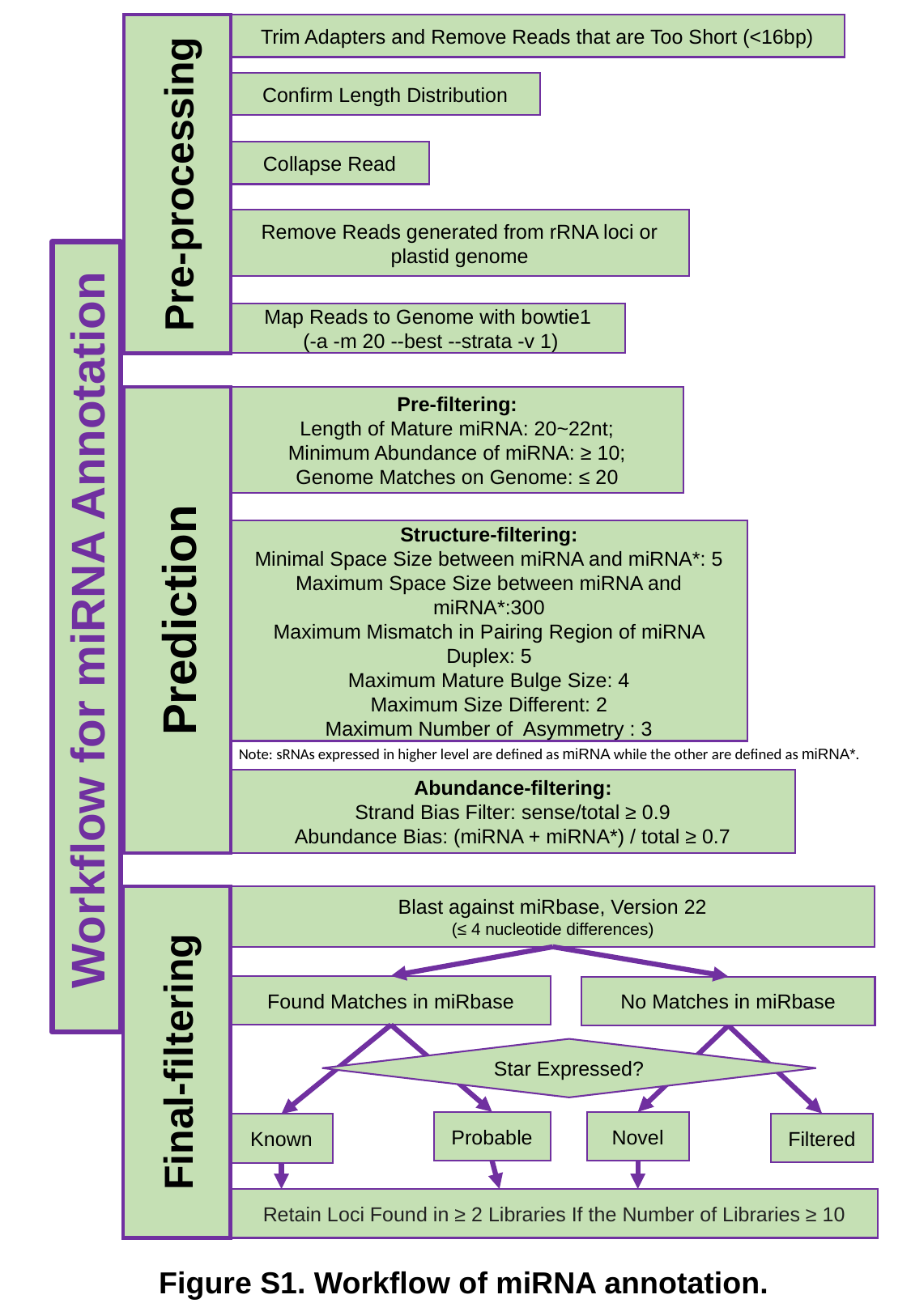

Pre-processing
Trim Adapters and Remove Reads that are Too Short (<16bp)
Confirm Length Distribution
Collapse Read
Remove Reads generated from rRNA loci or plastid genome
Map Reads to Genome with bowtie1
 (-a -m 20 --best --strata -v 1)
Prediction
Pre-filtering:
Length of Mature miRNA: 20~22nt;
Minimum Abundance of miRNA: ≥ 10;
Genome Matches on Genome: ≤ 20
Structure-filtering:
Minimal Space Size between miRNA and miRNA*: 5
Maximum Space Size between miRNA and miRNA*:300
Maximum Mismatch in Pairing Region of miRNA Duplex: 5
Maximum Mature Bulge Size: 4
Maximum Size Different: 2
Maximum Number of Asymmetry : 3
 Workflow for miRNA Annotation
Abundance-filtering:
Strand Bias Filter: sense/total ≥ 0.9
Abundance Bias: (miRNA + miRNA*) / total ≥ 0.7
Final-filtering
Blast against miRbase, Version 22
(≤ 4 nucleotide differences)
Found Matches in miRbase
No Matches in miRbase
Star Expressed?
Probable
Novel
Filtered
Known
Retain Loci Found in ≥ 2 Libraries If the Number of Libraries ≥ 10
Note: sRNAs expressed in higher level are defined as miRNA while the other are defined as miRNA*.
Figure S1. Workflow of miRNA annotation.

## Slide 2
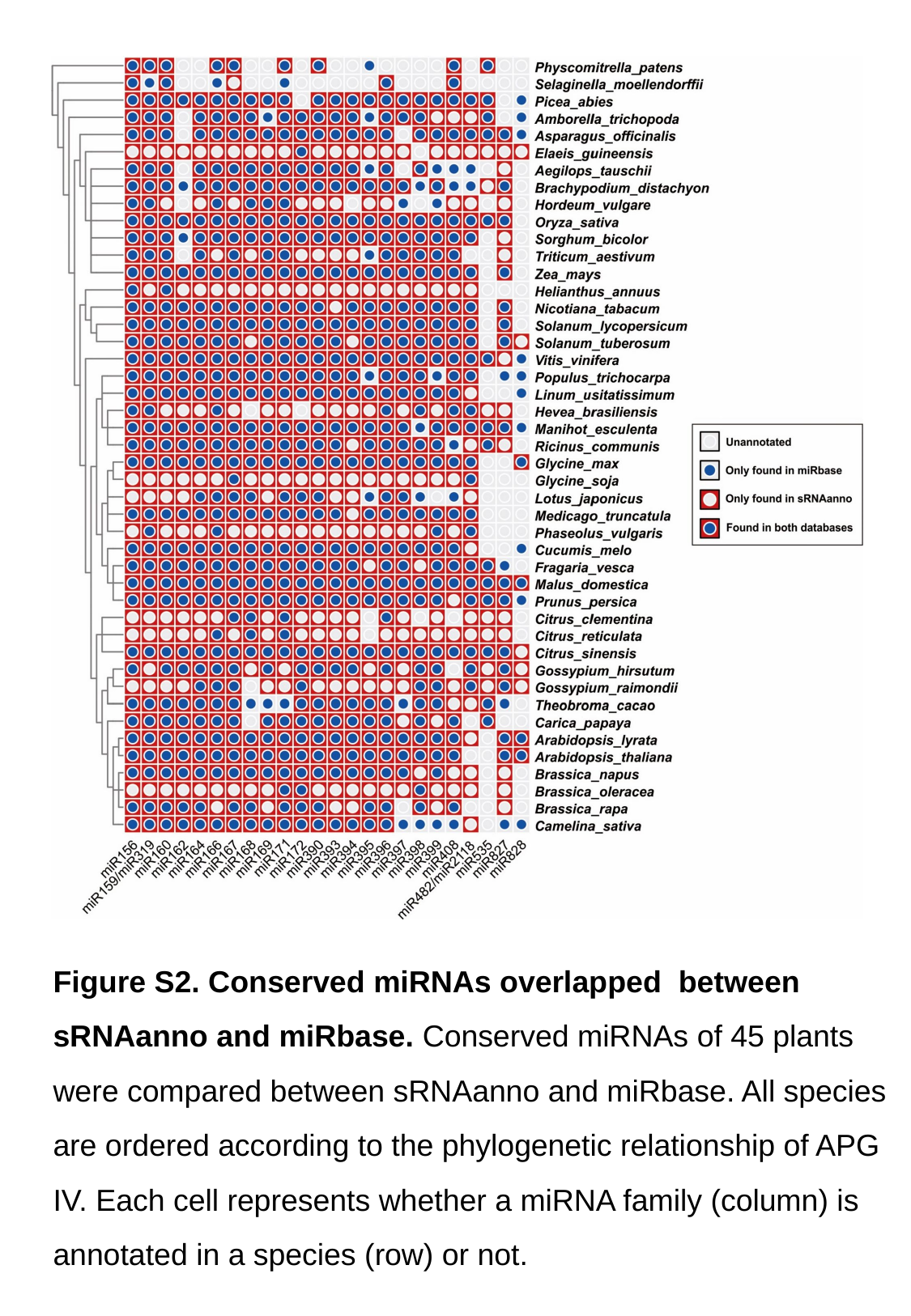

Figure S2. Conserved miRNAs overlapped between sRNAanno and miRbase. Conserved miRNAs of 45 plants were compared between sRNAanno and miRbase. All species are ordered according to the phylogenetic relationship of APG IV. Each cell represents whether a miRNA family (column) is annotated in a species (row) or not.

## Slide 3
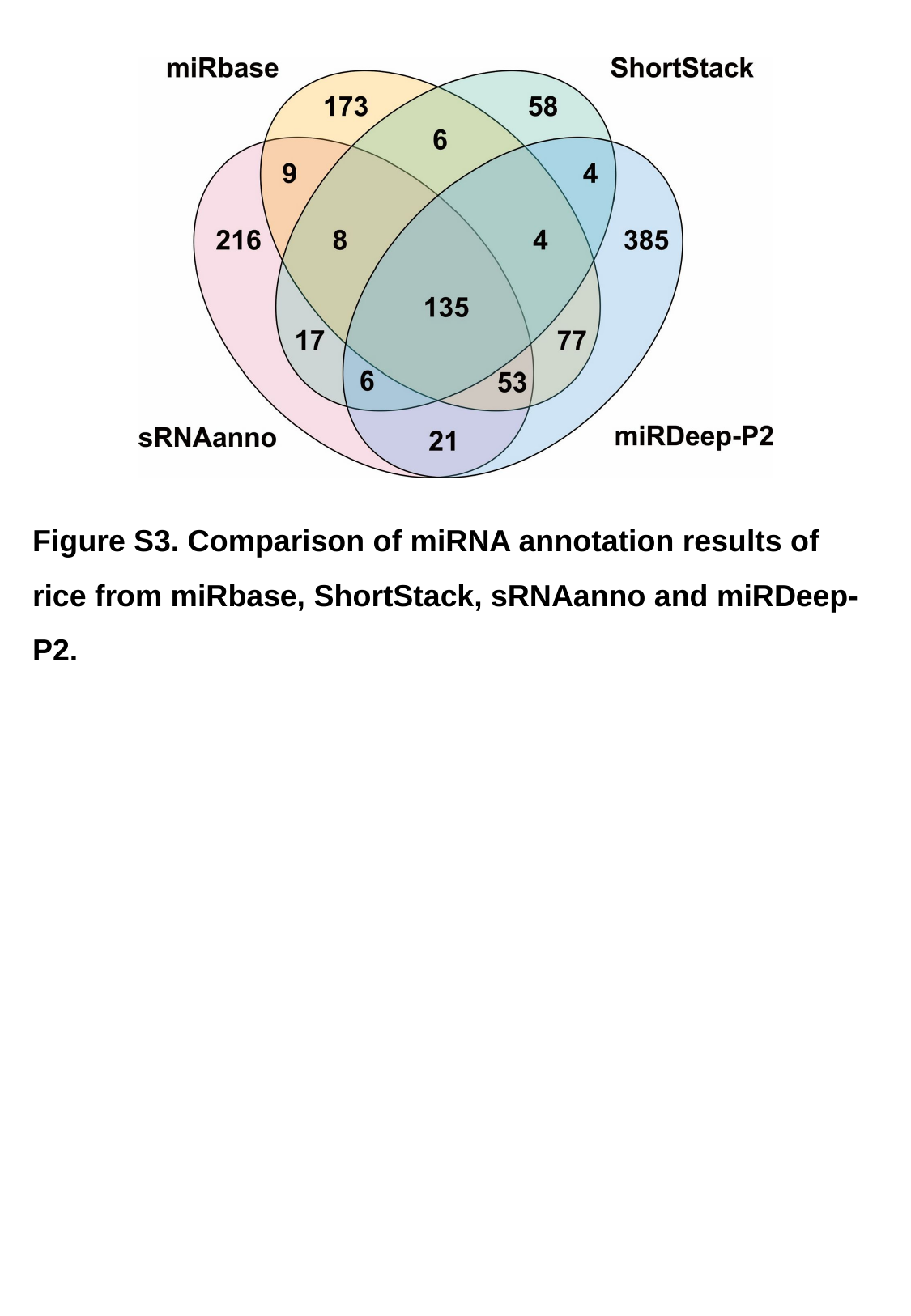

Figure S3. Comparison of miRNA annotation results of rice from miRbase, ShortStack, sRNAanno and miRDeep-P2.

## Slide 4
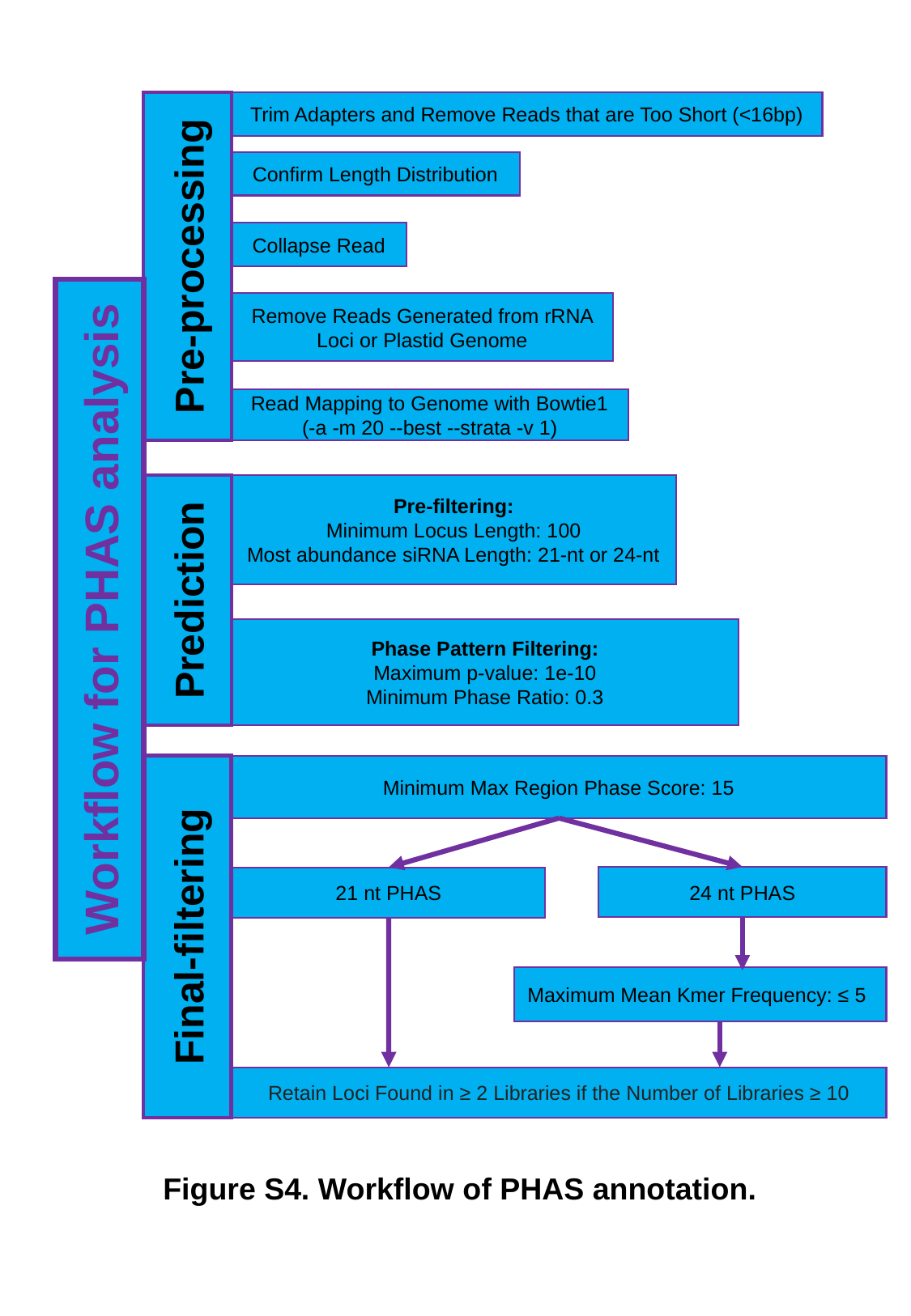

Pre-processing
Trim Adapters and Remove Reads that are Too Short (<16bp)
Confirm Length Distribution
Collapse Read
Workflow for PHAS analysis
Remove Reads Generated from rRNA Loci or Plastid Genome
Read Mapping to Genome with Bowtie1
(-a -m 20 --best --strata -v 1)
Prediction
Pre-filtering:
Minimum Locus Length: 100
Most abundance siRNA Length: 21-nt or 24-nt
Phase Pattern Filtering:
Maximum p-value: 1e-10
Minimum Phase Ratio: 0.3
Final-filtering
Minimum Max Region Phase Score: 15
24 nt PHAS
21 nt PHAS
Maximum Mean Kmer Frequency: ≤ 5
Retain Loci Found in ≥ 2 Libraries if the Number of Libraries ≥ 10
Figure S4. Workflow of PHAS annotation.

## Slide 5
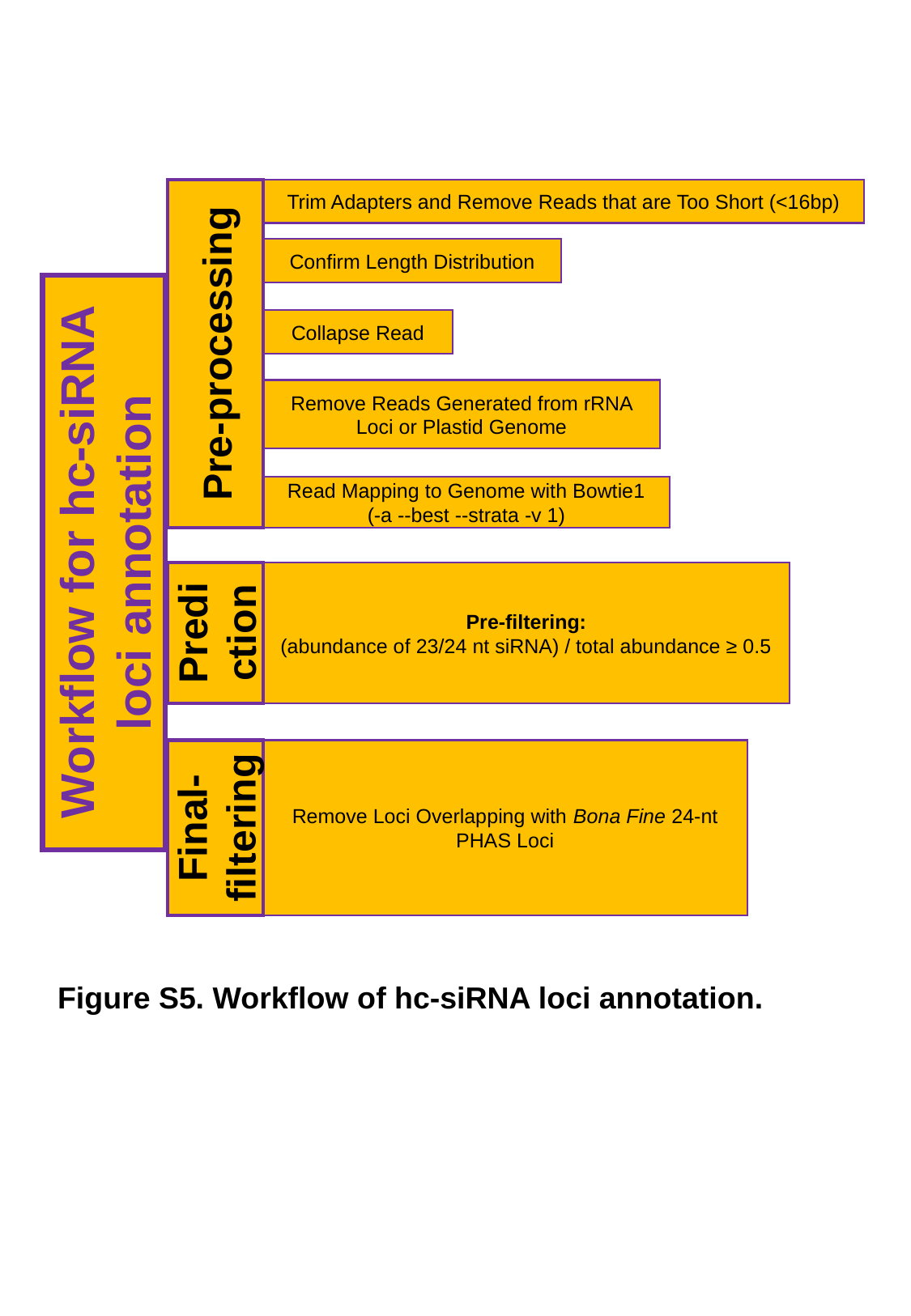

Pre-processing
Trim Adapters and Remove Reads that are Too Short (<16bp)
Confirm Length Distribution
Workflow for hc-siRNA loci annotation
Collapse Read
Remove Reads Generated from rRNA Loci or Plastid Genome
Read Mapping to Genome with Bowtie1
(-a --best --strata -v 1)
Prediction
Pre-filtering:
(abundance of 23/24 nt siRNA) / total abundance ≥ 0.5
Remove Loci Overlapping with Bona Fine 24-nt PHAS Loci
Final-filtering
Figure S5. Workflow of hc-siRNA loci annotation.

## Slide 6
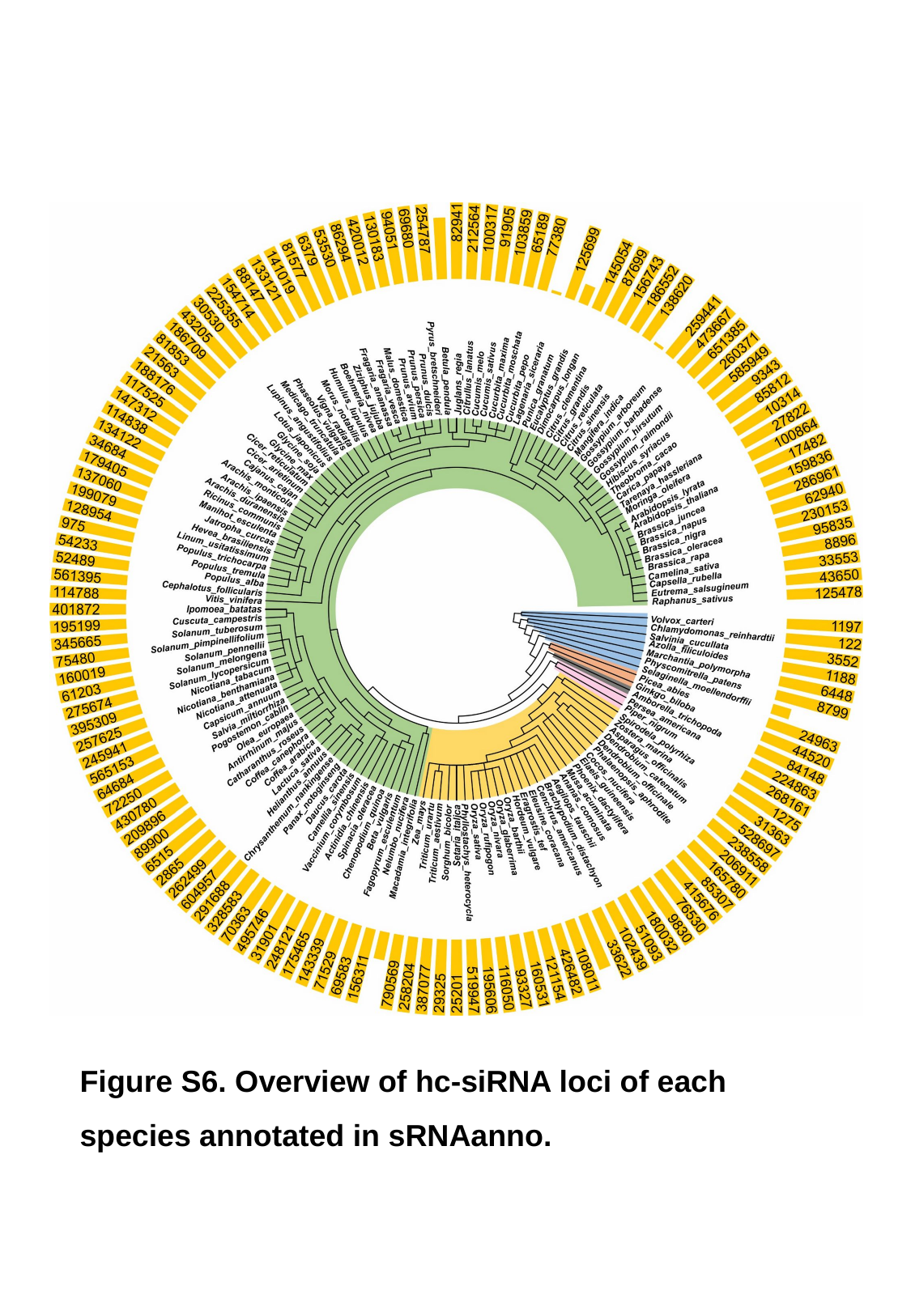

Figure S6. Overview of hc-siRNA loci of each species annotated in sRNAanno.
